# Ferroelectric-like Behavior Originating from Oxygen Vacancy Dipoles in Amorphous Film for Non-volatile Memory

**DOI:** 10.1186/s11671-020-03364-3

**Published:** 2020-06-22

**Authors:** Yue Peng, Genquan Han, Fenning Liu, Wenwu Xiao, Yan Liu, Ni Zhong, Chungang Duan, Ze Feng, Hong Dong, Yue Hao

**Affiliations:** 1 State Key Discipline Laboratory of Wide Band Gap Semiconductor Technology, School of Microelectronics, Xidian University, Xi’an, 710071 China; 2 Key Laboratory of Polar Materials and Devices, Ministry of Education, East China Normal University, Shanghai, 200241 China; 3 Key Laboratory of Photoelectronic, Thin Film Devices and Technology of Nankai University, Tianjin, 300071 China

**Keywords:** Amorphous, Al_2_O_3_, Ferroelectric, Memory, Oxygen vacancy dipole, Non-volatile field-effect transistor

## Abstract

Traditional ferroelectric devices suffer a lack of scalability. Doped HfO_2_ thin film is promising to solve the scaling problem but challenged by high leakage current and uniformity concern by the polycrystalline nature. Stable ferroelectric-like behavior is firstly demonstrated in a 3.6-nm-thick amorphous Al_2_O_3_ film. The amorphous Al_2_O_3_ devices are highly scalable, which enable multi-gate non-volatile field-effect transistor (NVFET) with nanometer-scale fin pitch. It also possesses the advantages of low process temperature, high frequency (~GHz), wide memory window, and long endurance, suggesting great potential in VLSI systems. The switchable polarization (*P*) induced by the voltage-modulated oxygen vacancy dipoles is proposed.

## Background

Ferroelectric random access memory (FeRAM) based on conventional perovskite ferroelectrics (e.g., PZT) has been one of the commercial non-volatile memories (NVMs) [1], although it cannot be scaled and not CMOS-compatible. Ferroelectricity was widely observed in a variety of different materials, such as porcine aortic walls [2], Sb_2_S_3_ nanowires [3], GaFeO_3_ film [4], doped poly-HfO_2_ films [5], nanocrystalline hydroxyapatite films [6], and LaAlO_3_-SrTiO_3_ film [7]. Among these materials, doped-HfO_2_ films have attracted special interests for the NVM application due to their CMOS process compatibility. But the polycrystalline structure is inevitable to generate ferroelectricity in doped-HfO_2_, which brought obstacles for device application to overcome as follows: 1) it is incompatible with the gate-last processing with regard to the thermal budget of 500 °C required to form orthorhombic crystal phases [8]; 2) power consumption is induced from undesired leakage current along the grain boundaries, which increases exponentially along with the scaling down of ferroelectric thickness. Recently, a theoretical study proposed that the additional ferroelectricity in thick poly-HfO_2_ (>5 nm) can come from the long-range correlations in the assembly of electric dipoles created by oxygen vacancies [9]. The defect charge trapping/detrapping mechanism was observed to produce the ferroelectric-like behavior in a 5-nm-thick amorphous Al_2_O_3_ for a multi-state memory, which, however, suffers from a very low trapping/detrapping frequency (e.g., ~500 Hz) [10].

In this work, stable ferroelectric-like behavior is demonstrated in a 3.6-nm-thick amorphous Al_2_O_3_ film, where the switchable polarization (*P*) is proposed to be induced by the voltage-modulated oxygen vacancy dipoles. The amorphous Al_2_O_3_ film possesses the advantages of low process temperature and the operating frequency up to ~GHz, which enable multi-gate non-volatile field-effect transistor (NVFET) with nanometer-scale fin pitch. Al_2_O_3_ NVFET memory with a 100-ns pulse width program/erase (P/E) voltages and over 10^6^ P/E cycles endurance is demonstrated. The effects of electrodes and film thickness on the *P* in Al_2_O_3_ capacitors are also investigated. The amorphous non-volatile devices show a promising future in VLSI memories.

## Methods

**Fig. 1:**
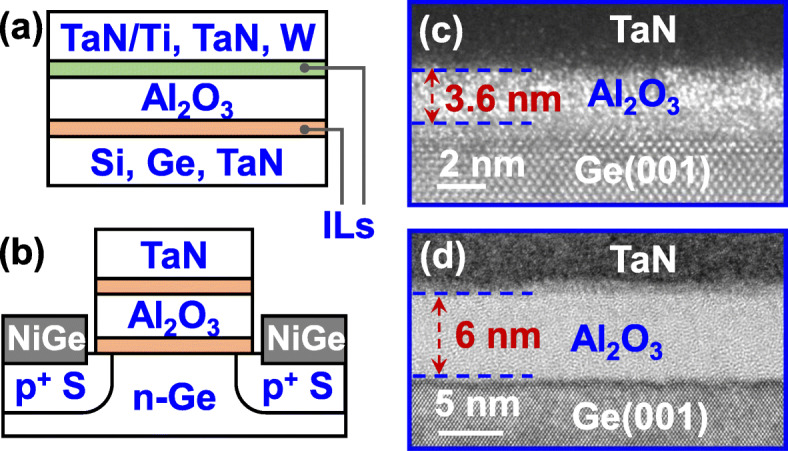
Schematics of the fabricated **a** Al_2_O_3_ capacitors with various electrodes and **b** Al_2_O_3_ NVFET. **c** and **d** HRTEM images of the fabricated TaN/Al_2_O_3_/Ge stacks with different *t*_AlO_, showing the amorphous Al_2_O_3_ films after an RTA at 350 °C

Amorphous Al_2_O_3_ films were grown on Si(001), Ge(001), and TaN/Si substrates by atomic layer deposition (ALD). TMA and H_2_O vapor were used as the precursors of Al and O, respectively. During the deposition, the substrate temperature was maintained at 300 °C. Different top metal electrodes, including TaN/Ti, TaN, and W, were deposited on Al_2_O_3_ surfaces by reactive sputtering. Capacitors with different electrodes were fabricated by lithography patterning and dry etching. Rapid thermal annealing (RTA) at 350 °C for 30 s was performed. NVFETs with TaN/Al_2_O_3_ gate stack were fabricated on Ge(001). After gate formation, source/drain (S/D) regions were implanted by BF_2_^+^ with a dose of 1 × 10^15^ cm^-2^ and an energy of 20 keV, and 20 nm-thick nickel S/D metal electrodes were then formed by lift-off process. Figure 1a and b shows the schematics of the fabricated Al_2_O_3_ capacitor and the p-channel NVFET. There is an interfacial layer (IL) between the electrode and the Al_2_O_3_ film. Figure 1c and d show the high-resolution transmission electron microscope (HRTEM) images of the TaN/Al_2_O_3_/Ge stacks with different amorphous Al_2_O_3_ thicknesses (*t*_AlO_) after an RTA at 350 °C.

## Results and Discussion

**Fig. 2:**
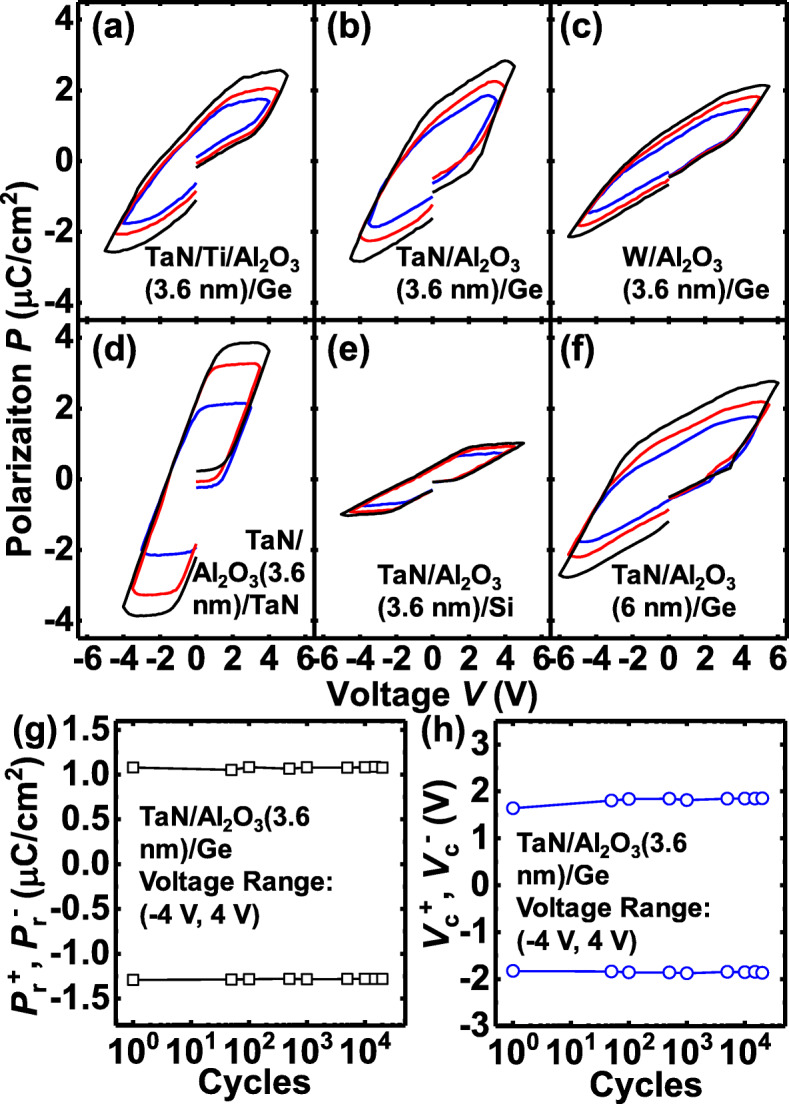
Measured *P* vs. *V* characteristics of the Al_2_O_3_ capacitors with different electrodes. **a**, **b**, and **c** showing the  *P*-*V* curves of TaN/Ti/Al_2_O_3_/Ge, TaN/Al_2_O_3_/Ge, and W/Al_2_O_3_/Ge, respectively, with a 3.6-nm *t*_AlO_. **d** and **e** showing that the *P*_sat_ is enhanced(reduced) by using TaN(Si) as the bottom electrode instead of Ge. **f** TaN/Al_2_O_3_(6 nm)/Ge capacitor has a higher *V*_c_ and a similar *P*_sat_ compared to the 3.6-nm-thick device in **b**. **g** and **h** Endurance measurements showing no degradation of *P*_r_ and *V*_c_ observed after 10^4^ sweeping cycles for a TaN/Al_2_O_3_(3.6 nm)/Ge capacitor

Figure 2 shows the measured *P* vs*.* voltage *V* characteristics for the amorphous Al_2_O_3_ capacitors with different *t*_AlO_ and various top and bottom electrodes. The measurement frequency is 1 kHz. As shown in Fig. 2a–c, with a fixed 3.6 nm of *t*_AlO_, TaN/Al_2_O_3_/Ge capacitor achieves a higher saturation *P* (*P*_sat_) compared to the devices with TaN/Ti and W top electrodes. The ferroelectric-like behavior is strongly correlated with interfaces, and it is proposed that the formation of TaAlO_*x*_ IL between TaN and Al_2_O_3_ produces more oxygen vacancies, contributing to a stronger switching *P*, compared to the TiAlO_*x*_ and WAlO_*x*_ ILs. *P-V* curves in Fig. 2d indicate that TaN/Al_2_O_3_/TaN capacitor has a much higher *P*_sat_ in comparison with TaN/Al_2_O_3_/Ge, which is attributed to the fact that dual TaAlO_*x*_ ILs provide higher oxygen vacancy concentration. While *P*_sat_ is significantly lower from that with Si bottom electrode (Fig. 2e), compared with the Ge electrode. This result indicates that Al_2_O_3_/Si interface quality is better, i.e., fewer oxygen vacancies, compared to that from the device based on Ge substrate. Figure 2f shows the *P-V* curves of a TaN/Al_2_O_3_(6 nm)/Ge capacitor, exhibiting a higher *V*_c_ and an almost identical *P*_sat_ as compared to that from the device with 3.6 nm of Al_2_O_3_ film in Fig. 2b. It is noted that the reason for the unclosed *P*-*V* loops is because a leakage indeed exists. It was reported that the large offset at an electric field of zero always occurred with a large field, and it always disappeared gradually with the smaller sweeping range of *V* [11, 12].

Figure 2g and h show the extracted evolution of the positive and negative remnant *P* (*P*_r_) and coercive *V* (*V*_c_) values, respectively, over 10^4^ sweeping cycles for a TaN/Al_2_O_3_/Ge capacitor. No wake-up, imprint, or fatigue effect is observed. *V*_c_ of the device is ~1.8 V, indicating that the *E* in the Al_2_O_3_ film is 4~6 MV/cm and in the ILs can exceed 8 MV/cm, which is high enough to drive the oxygen vacancies [13, 14]. *P*_sat_ of the devices ranges from 1 to 5 μC/cm^2^, corresponding to a reasonable oxygen vacancy concentration in the range 3~15×10^12^ cm^-2^ assuming they have charge of plus two.

**Fig. 3:**
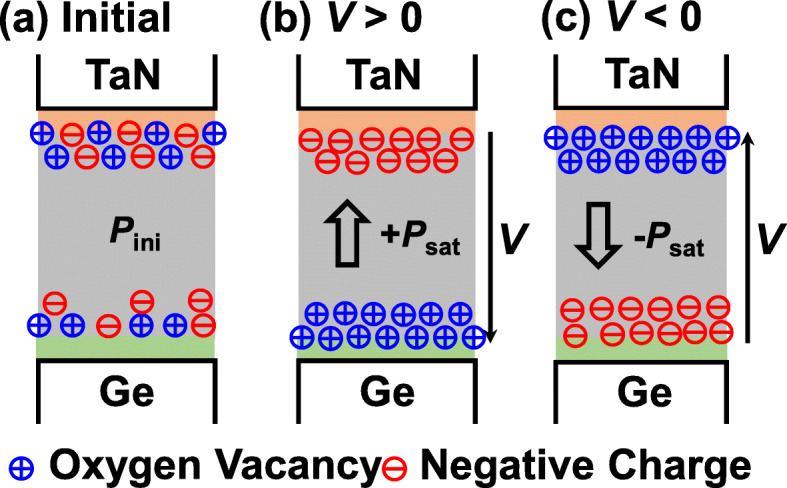
Schematics of the mechanism for ferroelectric-like behavior in Al_2_O_3_ capacitors. Switchable *P* is due to the migration of oxygen vacancies and negative charges to form dipoles

**Fig. 4:**
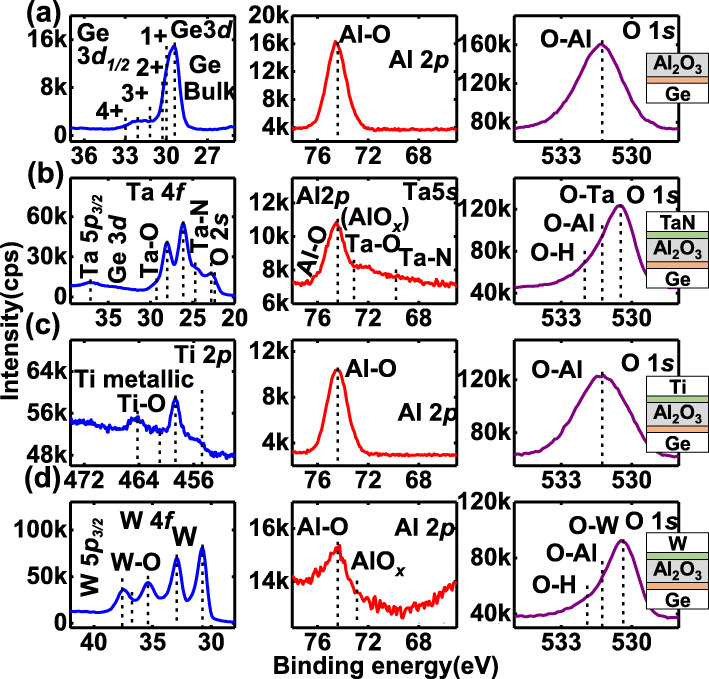
Core level XPS spectra of **a** Al_2_O_3_/Ge, **b** TaN/Al_2_O_3_/Ge, **c** Ti/Al_2_O_3_/Ge, and **d** W/Al_2_O_3_/Ge samples

The underlying mechanism for ferroelectric-like behavior associated with oxygen vacancies in Al_2_O_3_ devices is discussed. The migration of the voltage-driven oxygen vacancies has been widely demonstrated in resistive random-access memory devices [15, 16]. Figure 3 shows the schematics of the switchable *P* in TaN/Al_2_O_3_/Ge, which originates from the segregation of voltage-modulated oxygen vacancies and negative charges to form the electrical dipoles. It is reasonable to infer that the movable oxygen vacancies mainly arise from the formation of TaAlO_*x*_ IL and are located in the vicinity of the top interface at the initial state (Fig. 3a). Figure 3b and c  indicate how the positive and negative *P* are formed, respectively, with the modulation of the oxygen vacancy and negative charge dipoles under the applied voltage. X-ray photoelectron spectra (XPS) of Al_2_O_3_/Ge and (Ti, TaN, and W)/Al_2_O_3_/Ge samples are measured and shown in Fig. 4). For all the metal/Al_2_O_3_/Ge samples, there is a metal oxide IL formed between metal and Al_2_O_3_, which are proposed to be the reservoir of oxygen ions and vacancies, which is consistent with Ref. [17].

**Fig. 5:**
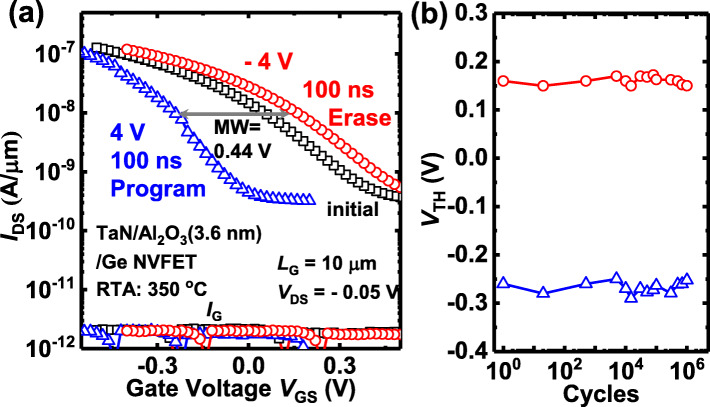
**a** Measured *I*_DS_-*V*_GS_ curves of a 3.6-nm-thick Al_2_O_3_ NVFET for the initial and two polarization states. An MW of 0.44 V is obtained. **b** Endurance measurement demonstrates that no MW degradation is observed after 10^6^ P/E cycles

To characterize the electrical performance of Al_2_O_3_ NVFET as NVM, program (erase) operation is achieved by applying positive (negative) voltage pulses to the gate, to raise (lower) its threshold voltage (*V*_TH_). Figure 5a shows how the linear-region transfer characteristics of the Al_2_O_3_ NVFET shift relative to the initial *I*_DS_-*V*_GS_ curve measured with ±4 V program (erase) voltages with 100 ns pulse width. Here, *V*_TH_ is defined as a *V*_GS_ at 100 nA⋅W/L, and MW is defined as the maximum change in *V*_TH_. The Al_2_O_3_ NVFET obtains an MW of 0.44 V, though amorphous Al_2_O_3_ film has smaller *P*_r_ than the reported doped HfO_2_ films [5, 8]. It is noted that the high operating frequency up to 10 MHz of Al_2_O_3_ NVFET memory, which is indicative of that switchable *P* in Al_2_O_3_ originates from the migration of voltage-driven oxygen vacancy to form dipoles, not from defects charge trapping/detrapping. Alternating program and erase pulses were applied to the Al_2_O_3_ devices to further study the device endurance. Figure 5b shows the plots of *V*_TH_ vs*.* P/E cycle number, suggesting a stable MW can be maintained without a significant degradation over 10^6^ P/E cycles for a 3.6-nm-thick Al_2_O_3_ NVFET.

Notably, the ferroelectric-like behavior observed in the amorphous Al_2_O_3_ devices can be extended to the universal amorphous oxides, e.g., hafnium oxide (HfO_2_) and zirconium oxide (ZrO_2_).

## Conclusions

Stable ferroelectric-like behavior is first realized in capacitors with a thin amorphous Al_2_O_3_ insulator. Switchable *P* in amorphous Al_2_O_3_ capacitors is demonstrated by *P-V* loops and NVFET test. The ferroelectric-like behavior is proposed to be originating from the interface oxygen vacancies and ions dipoles. The 3.6-nm-thick Al_2_O_3_ NVFET achieves an MW of 0.44 V and over 10^6^ cycle endurance under ±4 V at 100 ns P/E condition. All in all, this work opened a new world for amorphous oxide ferroelectric devices, which are promising for multi-gate (fin-shaped, nanowire, or nanosheet) NVFETs with potentially nano-scaled fin pitch in VLSI systems.

## Data Availability

The datasets supporting the conclusions of this article are included in the article.

## Abbreviations

Al_2_O_3_: Aluminum oxide
ALD: Atomic layer deposition
BF_2_^+^: Boron fluoride ion
*E*_c_: Coercive electric field
Ge: Germanium
GeO_*x*_: Germanium oxide
HRTEM: High-resolution transmission electron microscope
*I*_DS_: Drain current
MOSFETs: Metal-oxide-semiconductor field-effect transistors
MW: Memory window
Ni: Nickel
NVFET: Non-volatile field-effect transistor
*P*_r_: Remnant polarization
*P*_sat_: Saturation polarization
RTA: Repaid thermal annealing
TaAlO_*x*_: Tantalum aluminum oxide
*t*_AlO_: Aluminum oxide thickness
TaN: Tantalum nitride
*V*_GS_: Gate voltage
*V*_TH_: Threshold voltage
XPS: X-ray photoelectron spectra
